# Characteristics and Epidemiology of Megaprostheses Infections: A Systematic Review

**DOI:** 10.3390/healthcare12131283

**Published:** 2024-06-27

**Authors:** Luigi Cianni, Francesco Taccari, Maria Beatrice Bocchi, Giulia Micheli, Flavio Sangiorgi, Antonio Ziranu, Massimo Fantoni, Giulio Maccauro, Raffaele Vitiello

**Affiliations:** 1 Dipartimento di Scienze dell’invecchiamento, Ortopediche e Reumatologiche, Unità Operativa Complessa di Ortopedia e Traumatologia, Fondazione Policlinico Universitario A. Gemelli IRCCS, 00168 Rome, Italy; luigi_cianni@libero.it (L.C.); giulio.maccauro@policlinicogemelli.it (G.M.); lele.vitiello@gmail.com (R.V.); 2 Dipartimento di Sicurezza e Bioetica—Sezione di Malattie Infettive, Università Cattolica del Sacro Cuore, 00168 Rome, Italy; micheli93giulia@gmail.com (G.M.); sangiorgi.flavio@gmail.com (F.S.); antonio.ziranu@policlinicogemelli.it (A.Z.); massimo.fantoni@policlinicogemelli.it (M.F.); 3 Dipartimento di Scienze Mediche e Chirurgiche, Unità Operativa Complessa di Malattie infettive, Fondazione Policlinico Universitario A. Gemelli IRCCS, 00168 Rome, Italy; francesco.taccari@policlinicogemelli.it; 4 Ospedale Isola Tiberina-Gemelli Isola, 00186 Rome, Italy

**Keywords:** megaprostheses, resection arthroplasty, infection

## Abstract

Background: Megaprostheses were first employed in oncological orthopedic surgery, but more recently, additional applications have arisen. These implants are not without any risks and device failure is quite frequent. The most feared complication is undoubtedly the implants’ infection; however, the exact incidence is still unknown. This systematic review aims to estimate in the current literature the overall incidence of megaprosthesis infections and to investigate possible risk/protective factors. Methods: We conducted a systematic search for studies published from July 1971 to December 2023 using specific keywords. To be included, studies needed to report either the megaprosthesis anatomical site, and/or whether the megaprosthesis was coated, and/or the surgical indication as oncological or non-oncological reasons. Results: The initial literature search resulted in 1281 studies. We evaluated 10,456 patients and the overall infection rate was 12%. In cancer patients, the infection rate was 22%, while in non-oncological patients, this was 16% (trauma 12%, mechanical failure 17%, prosthetic joint infections 26%). The overall infection rates comparing coated and uncoated implants were 10% and 12.5%, respectively. Conclusions: The number of megaprosthesis implants is increasing considerably. In traumatological patients, the infection rate is lower compared to all the other subgroups, while the infection rate remains higher in the cancer patient group. As these devices become more common, focused studies exploring epidemiological data, clinical outcomes, and long-term complications are needed to address the uncertainties in prevention and management.

## 1. Introduction

Massive long bone defects pose significant challenges for reconstruction in the orthopedic field. Various techniques and strategies have been adopted to treat these bone defects, such as autograft and allogeneic bone grafting, bone transport, and the use of standard prostheses and megaprostheses (MP) [1]. Megaprostheses, also referred to as tumor endoprostheses, are systems that allow special segmental bone and joint replacement, which were initially developed for lower limb and then upper limb salvage. First employed in oncological orthopedic surgery in the 1960s [2], megaprostheses, along with the advent of adjuvant therapies, have dramatically changed the management of bone tumors, which previously condemned patients to limb amputation. Since the 1990s, megaprostheses have become the gold standard for reconstruction after the resection of primary malignant bone tumors. Occasionally, further oncological indications have come into place, such as reconstruction after the eradication of locally aggressive benign bone tumors, malignant soft tissue tumors, or bone metastasis [3,4]. More recently, additional applications have arisen for megaprostheses implants as a last resort for revision arthroplasty in selected cases, where extensive bone loss or poor bone quality jeopardizes the success of conventional joint arthroprosthesis, or for trauma/revision trauma surgery with severe soft tissue damage, severely comminuted fractures, or similar bone quality concerns [5,6]. As expected, however, the implant of a megaprostheses is not without any risks, and device failure is more frequent than in other primary joint arthroplasties [7,8]. Recently, Henderson et al. [9] proposed a classification system for megaprostheses failure. The latter can be divided into mechanical causes, such as soft tissue failure, aseptic loosening, and structural failure, and nonmechanical, such as infection and tumor progression. Mechanical challenges such as dislocation are quite common: the rate of hip dislocation after proximal femur replacement ranges from 6% to 42% [10,11]. Another major challenge lies in the recovery of good joint function, especially when the knee is involved. Scar tissue, joint problems from previous deformities or concomitant degeneration, and muscle loss with loss of contractile function are common in patients undergoing megaprostheses placement and have a deep impact in joint mobility recovery. Failure can also originate from the low quality of superficial soft tissue coverage, which can be compromised following trauma, septic conditions, or recurrent surgeries [12], or by adjuvant therapy in oncologic diseases, such as radiotherapy [13]. When the implant is placed for tumors, recurrence and progression are other possible causes of implant failure and reduced survival [9]. In addition, one of the most feared complications is megaprostheses infection, which is even more frequent than periprosthetic infections according to multiple studies. However, the exact incidence of periprosthetic infection in patients with megaprostheses is still unknown and may range from 3% [14] to higher than 30% [9,15]. Infections are facilitated by a longer surgery time and wider soft tissue dissection compared to other orthopedic procedures, along with a usually more immunocompromised host who may need multiple revision surgeries [16]. Although indications for the implantation of megaprostheses are increasing, this type of prostheses is still quite rare. Therefore, the exact epidemiology and incidence rate of megaprostheses infection are largely unknown due to a lack of literature. To date, it is also not yet known whether there are specific risk factors that may negatively influence the risk of infection for these implants. This systematic narrative review first and foremost aims to estimate in the current literature the overall incidence of megaprostheses infections. Furthermore, it aims to investigate possible risk/protective factors with respect to the onset of infections such as the impact of the anatomical location, the indications for placement, and the presence/absence of implant coating. Greater clarity in the matter could help to establish preventive measures and to give clues in terms of management and outcomes.

## 2. Materials and Methods

**Figure 1:**
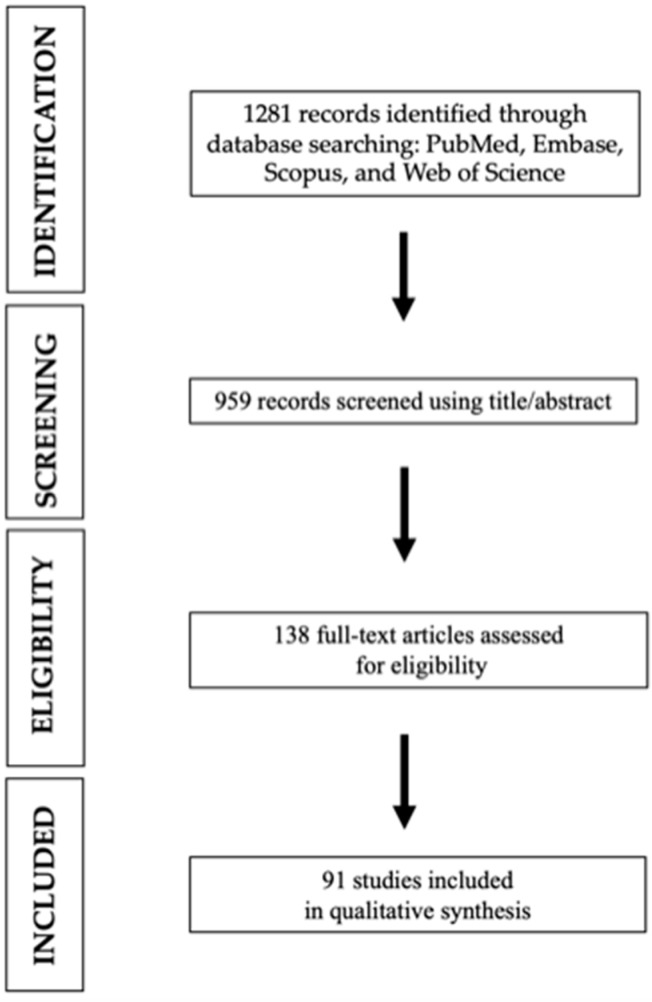
Search and literature selection.

We conducted a comprehensive systematic search on four databases (PubMed, Embase, Scopus, and Web of Science) using the search keywords ((megaprostheses) OR (megaprosthesis) OR (resection arthroplasty) AND (infection)). No restrictions were applied to the publication dates. Therefore, the research included studies published from July 1971 until December 2023. The bibliographies of the selected studies were manually searched to identify additional papers not found through the ordinary search. The titles of the journal, authors’ names, and supporting institutions were known throughout the whole process. This systematic review was conducted according to the latest version of Preferred Reporting Items for Systematic Reviews and Meta-analyses (PRISMA) [17], as reported in Figure 1. The 27-item PRISMA checklist of this systematic review can be found in the Supplementary Materials. This study was not registered in any protocol database; therefore, there is no registration number. Inclusion criteria were specified as follows: studies published in Italian, English, or French, describing the number of infected implants out of the total number of megaprostheses implanted. Moreover, studies had to report either the megaprostheses anatomical site (total femur, proximal tibia, distal femur, proximal femur, proximal humerus, custom megaprosthesis after hemipelvectomy, and other), and/or whether the megaprostheses were coated, and/or the surgical indication as oncological or non-oncological reasons. Exclusion criteria were established as follows: review articles (systematic or narrative), meta-analysis, letters, case reports, notes, conference papers, editorials, and conference abstracts; cadaveric and animal studies; full text not available; and finally, articles published in any language other than the previously stated ones. The primary outcome is to determine the overall incidence of infection per implanted megaprostheses. The secondary outcome is to establish the occurrence of megaprosthesis infection per implanted megaprostheses categorized by anatomy (total femur, proximal tibia, distal femur, proximal femur, proximal humerus, custom megaprostheses after hemipelvectomy), coating (presence or absence), and reason for implantation (oncological and non-oncological, with the latter further divided into trauma, periprosthetic joint infections (PJIs), and mechanical failure). Abstracts and full texts were independently screened by three authors (L.C., F.S., and G.M.), and any arising conflict was solved by consensus with a fourth author (F.T.). All the selected studies were retrospectively analyzed by two authors (M.B.B. and R.V.) who then extracted and entered the data in an Excel worksheet. The collected data included authors’ list, year of publication, and the number of infected prostheses out of the total number of megaprostheses implanted. In addition, the implanted and then infected megaprostheses were specifically studied according to the anatomical district involved, the presence or absence of coating of the megaprostheses, and indication for megaprostheses implantation. Lastly, the data sheet was reviewed by three authors (A.Z., M.F., and G.M.) who agreed and validated the extracted data.

## 3. Results

### 3.1. Search and Literature Selection

The initial literature search resulted in 1281 studies. After reviewing all the abstracts, the restricted research retrieved 959 studies. Once duplicates were removed and the articles were screened for inclusion and exclusion criteria, 138 studies remained, and full texts were assessed for eligibility (Figure 1). Finally, a total of 91 articles were included in this systematic review [15,18,19,20,21,22,23,24,25,26,27,28,29,30,31,32,33,34,35,36,37,38,39,40,41,42,43,44,45,46,47,48,49,50,51,52,53,54,55,56,57,58,59,60,61,62,63,64,65,66,67,68,69,70,71,72,73,74,75,76,77,78,79,80,81,82,83,84,85,86,87,88,89,90,91,92,93,94,95,96,97,98,99,100,101,102,103,104,105,106,107].

### 3.2. Study Characteristics

We evaluated 10,456 patients who had undergone limb reconstruction with modular megaprosthesis. The infection rate was 12% (1277/10,456). Our results are summarized in Table 1.

### 3.3. The Initial Diagnosis

Most patients had an indication to implant a modular megaprosthesis after tumor resection (4283/10,456, 41%) including either primary tumors or metastatic disease. In cancer patients, the infection rate was 22% (941/4283). Among all patients who instead had a megaprosthesis implanted in a non-oncological setting, the infection rate was 16% (221/1377). More specifically, 652 modular megaprostheses were implanted for significant bone loss due to major trauma, failed osteosynthesis with a nonunion, or even periprosthetic fractures. The infection rate in this group of patients was 12% (76/652). Furthermore, 142 megaprostheses were implanted in the case of mechanical failure, and among them, 24 later became infected with an infection rate of 17%. Finally, 318 modular implants were used in periprosthetic joint infections (PJIs) and the re-infection rate was found to be 26% (84/318).

### 3.4. Anatomical Classification

Among all the modular megaprostheses included in the study, 7766 were implanted in the lower limbs and 265 in the upper limbs. The overall infection rates were 15% (1155/7766) and 10% (27/265), respectively. Among the lower limb implants, 139 were total femur replacements of which 39 became infected (28%), 5780 were either proximal tibia or distal femur replacements with 885 ending up being infected (15%), and finally, 1402 were proximal femur replacements with an infection rate of 13% (187/1402). Among the 142 megaprostheses of proximal humerus included, 13 became infected (9%). Hemipelvectomy and reconstruction with a custom megaprosthesis was performed in 21 patients and among them 8 became infected (38%).

### 3.5. Coated vs. Uncoated

Among the 10,456 modular megaprostheses implanted, 1407 were silver coated and 9049 were uncoated. The overall infection rates comparing the two groups were 10% (141/1407) and 12.5% (1135/9049), respectively.

## 4. Discussion

Megaprosthesis replacement was originally used for reconstructive treatment in limb salvage surgery following soft tissue and bone tumor resections [108]. To date, indications are becoming broader and megaprostheses are now used in the management of bone loss such as in severe trauma, periprosthetic fracture, and arthroplasty revision, or in the case of periprosthetic joint infection [109]. However, the patient selection in this type of surgery is crucial because a possible revision surgery could be technically challenging with a high clinical burden and low functional recovery, especially in the frail population [110,111]. Given its increasing frequency, orthopedic surgeons should be aware of the risk of infection of a megaprosthesis as this is a complication that can lead to adverse clinical and functional outcomes, and in some cases, even to the patient’s death [112].

As far as we know, no recent epidemiological reviews in the literature have fully addressed this problem. We recruited 91 articles for a total of 10,456 patients with different features of indications, anatomy, and coating. Among these, the overall megaprosthesis infection rate was 12%.

Our data showed different infection rates based on the different initial diagnosis.

Our study showed that there exists an important difference in terms of the infection rate between cancer patients (22%) and non-cancer patients (16%). The poor general clinical conditions of an oncological patient may explain such data. A recent review by Gonzalez MR et al. identified several modifiable and unmodifiable risk factors such as chemotherapy, radiation therapy, immunosuppression and sufficient soft tissue coverage, operative time, and length of stay, which have to be considered when a cancer patient has to undergo surgery [113]. Previous studies indicated an incidence of infections of approximately 8% in megaprostheses implanted for oncological reasons, far different from our data [9]. Certainly, the increased use of these prostheses even only in the oncological context together with the increased survival of these patients leads to an increase in complications and thus also infections. On the other hand, Vayhsa et al. instead recruited studies focusing on the implantation of lower limb megaprostheses in non-cancer patients finding an infection rate of 18.5%, comparable to our findings [114]. We can therefore conclude that cancer patients with megaprostheses have a higher risk of implant infections compared to non-oncological patients. Concerning more specifically the non-oncological group, the infection rate in patients with megaprostheses implanted for traumatological indication is 12%, which is higher than expected. Indeed, recent findings by Sambri et al. [109] showed instead an incidence of 8.2% that is explainable, however, by a difference in the sample. Considering mechanical failure as an indication for megaprosthesis implants, the infection incidence rate was 17%. Studies on the same indication in the lower limb described a similar rate (18%) [49,113], while few data exist concerning the upper limb, describing very few cases of infection [114,115]. In the subgroup with PJI as the lead indication for megaprosthesis implants, the infection rate was 26%; higher than the other two subgroups, as expected, although in line with the existing literature [20].

Furthermore, our data showed different infection rates based on the anatomical site.

Lower limb megaprostheses demonstrated an infection rate of 15% (1155/7766 patients), compared to 10% (27/265) in the upper limb. Similar values were found by Schmidt-Braekling et al. who showed a lower limb infection rate higher than 17% [116]. However, the data in the literature are not unique. Indeed, Windagher et al. reported a rate of infection in patients treated with a megaprosthesis after a distal femur periprosthetic fracture that ranged from 6.6% after 1 year to 45% after a mean follow-up of 34 months [117].

Going into further detail, we found a higher rate of infection in knee megaprostheses compared to hip megaprostheses. These data can be explained both by the greater number of knee megaprostheses implanted and also because the knee is a most insidious anatomical site, which often does not provide adequate soft tissue coverage [118].

Finally, considering the outcomes for coated or uncoated megaprostheses, we found a higher infection rate for uncoated megaprostheses. Unfortunately, to date there are no univocal results in the literature on this matter. Not all studies included specified whether the megaprostheses were coated or uncoated; however, we assumed that when unspecified, the implants were uncoated. A total of 9049 patients had uncoated megaprostheses implanted with an infection rate of 12.5% (1135/9049 patients). The infection rate appears to decrease for coated implants such that in this group the infection rate was 10% (141/1407 patients). Lex JR et al. performed a review on the different outcomes between coated or uncoated megaprostheses, investigating major anti-bacterial coatings currently in use, including 11 studies: only 2 studies demonstrated better outcomes in coated megaprostheses compared to uncoated ones [115]. Furthermore, Fiore M et al. did not find any statistically significant difference between the outcomes in coated megaprostheses compared to uncoated ones, concluding that coated megaprostheses should only be used in selected cases [119]. No clear conclusions could be drawn on the difference of outcomes between coated and uncoated; however, our findings seem to suggest a potential protective role of coated prostheses as already mentioned by other authors.

Our paper has several limitations. This is a systematic review that was not registered. Furthermore, the articles’ quality has not been assessed. Moreover, although it was possible to extrapolate the number of implanted megaprostheses and the rate of infections in all included works, unfortunately, it was not always possible, for instance, to trace the reasons why the prosthesis was implanted. Therefore, such detailed tables were purposely included. In addition, due to the wide heterogeneity of the articles included in this review, it was not possible to assess the follow-up and then relate all our data to it. This is mostly because too often the follow-up was not mentioned or, unfortunately, was not relatable to the individual groups.

The present literature review focuses on the significantly increasing number of megaprostheses implantations and, accordingly, the increasing number of implant failures due to infections. To the best of our knowledge, there is no review in the literature that includes more than 10,000 patients undergoing megaprosthesis replacement. However, the large number of works examined, and the wide variability in the examined data and outcomes do not allow us to draw clear conclusions. In the future, the opportunity to identify not only predisposing but also protective factors with respect to megaprostheses will make it possible to develop standardized treatment algorithms to reduce the risks for patients in general, and specifically the infectious one. These would enable us to select patients and thus associate each one with a specific device and surgical approach to optimize treatment and outcomes.

## 5. Conclusions

In conclusion, the number of megaprosthesis implants is increasing considerably. While in the past megaprosthesis implants were exclusive to cancer patients, to date, they are increasing in number by increasing the indications. Nowadays, megaprostheses are mostly used for lower limb replacements, especially around the knee. Megaprostheses implanted as a consequence of severe trauma are increasing significantly, second only to cancer patients. In traumatological patients, the infection rate is lower than in both cancer patients and patients with PJI but also in patients with the mechanical failure of prostheses. Knee megaprostheses have a higher infection risk than hip megaprostheses, which can help the surgeon to decide on different perioperative management approaches for the hip and the knee. Coated implants have a slightly lower infection rate than uncoated implants and should be considered in patients at high risk of infection. As these devices become more common, focused studies exploring epidemiological data, clinical outcomes, and long-term complications are needed to address the uncertainties in prevention and management.

## Figures and Tables

**Table 1:** Infection rate in relation to principal diagnosis, anatomy, and implant characteristics.

Authors	Year of Publication	Megaprostheses	Infections	Infections/ Knee Replacement	Infections/ Hip Replacement	Infections/ Total Femur Replacement	Infections/ Shoulder Replacement	Infections/ Other	Infections/ Coated	Infections/ Uncoated	Infections/ Oncological	Infections/ Trauma	Infections/ PJI	Infections/ Mechanical Failure
Berger C et al. [24]	2021	116	35	20/52	7/30	0/3	4/15	4/16	-	35/116	25/116	-	-	-
Şirin E et al. [90]	2023	553	32	24/24	7/7	-	1/1	-	-	32/553	32/553	-	-	-
Khakzad T et al. [57]	2022	82	14	9/32	2/30	1/1	2/19	-	3/57	10/25	13/82	-	-	-
Gulia A et al. [44]	2023	14	10	10/14	-	-	-	-	-	10/14	10/14	-	-	-
Sacchetti F et al. [83]	2022	142	29	-	-	-	-	-	9/38	20/104	-	-	-	-
Asokan A et al. [23]	2022	14	14	5/5	6/6	3/3	-	-	-	14/14	-	7/7	3/3	4/4
Pala E et al. [75]	2022	187	13	9/77	0/38	-	4/50	-	8/118	5/69	13/180	0/7	-	-
Innocenti M et al. [54]	2023	444	19	17/17	2/2	-	-	-	-	19/444	19/444	-	-	-
Theil C et al. [98]	2021	568	58	44/445 *			14/123 **	-	58/568	-	58/568	-	-	-
Murphy EP et al. [68]	2021	72	12	12/77	-	-	-	-	-	12/72	12/72	-	-	-
Grandhi TSP et al. [43]	2021	26	6	6/26	-	-	-	-	6/26	-	6/26	-	-	-
Zoccali C et al. [107]	2021	86	6	-	-	-	-	-	0/43	6/43	-	-	-	-
Ogura K et al. [71]	2021	214	112	112/214	-	-	-	-	-	112/214	112/214	-	-	-
Gundavda MK et al. [45]	2020	35	35	26/26	9/9	-	-	-	-	35/35	35/35	-	-	-
Sambri A et al. [84]	2020	68	13	13/68	-	-	-	-	3/29	10/39	13/68	-	-	-
Mazaleyrat M et al. [66]	2020	161	25	25/161	-	-	-	-	-	25/161	25/161	-	-	-
Bischel OE et al. [26]	2020	45	1	-	1/45	-	-	-	-	1/45	1/45	-	-	-
Zhang HR et al. [106]	2020	177	21	21/77	-	-	-	-	-	21/177	21/177	-	-	-
Kamal AF et al. [55]	2020	19	2	2/19	-	-	-	-	-	2/19	2/19	-	-	-
Smolle MA et al. [92]	2020	57	11	6/26	5/31	-	-	-	-	11/57	-	-	-	-
Kamal AF et al. [55]	2019	8	1	1/8	-	-	-	-	-	1/8	1/8	-	-	-
Lam YL et al. [59]	2019	58	6	2/6	1/6	-	1/6	1/6	-	6/58	6/58	-	-	-
Sigmund IK et al. [89]	2018	621	83	70/70	12/12	1/1	-	-	-	83/621	83/621	-	-	-
Aebischer AS et al. [18]	2022	306	10	10/306	-	-	-	-	-	10/306	-	10/306	-	-
Apprich SR et al. [21]	2021	33	5	3/14	0/12	2/7	-	-	-	5/33	-	5/33	-	-
Vertesich K et al. [104]	2019	30	8	8/30	-	-	-	-	-	8/30	-	-	5/9	-
Sobol KR et al. [93]	2022	75	16	16/75	-	-	-	-	-	16/75	-	5/27	5/23	2/25
Logoluso N et al. [60]	2022	21	2	-	2/21	-	-	-	2/12	0/9	-	-	2/21	-
Sukhonthamarn K et al. [96]	2021	219	33	18/146	15/73	-	-	-	-	33/219	-	8/81	20/63	5/67
Smith EL et al. [91]	2020	42	10	9/29	1/13	-	-	-	-	10/42	-	2/13	6/19	2/10
Rajasekaran RB et al. [79]	2020	24	1	1/24	-	-	-	-	-	1/24	-	1/24	-	-
Döring K et al. [38]	2021	28	6	-	6/28	-	-	-	-	6/28	-	-	6/11	-
De Martino I et al. [36]	2019	31	3	-	3/31	-	-	-	-	3/31	-	3/31 ***		
Streitbuerger A et al. [95]	2019	99	11	-	11/99	-	-	-	6/64	5/35	11/99	-	-	-
Hardes J et al. [47]	2018	98	15	15/98	-	-	-	-	7/56	8/42	15/98	-	-	-
Holm CE et al. [50]	2018	50	6	-	-	-	-	-	-	6/50	6/50	-	-	-
De Gori M et al. [34]	2017	169	2	-	-	-	-	-	-	2/169	2/169	-	-	-
Yang P et al. [105]	2017	8	1	-	-	-	-	1/8	-	1/8	1/8	-	-	-
Toepfer A et al. [101]	2017	36	13	13/36	-	-	-	-	-	13/36	6/20	7/16 ***		
Piccioli A et al. [77]	2016	30	5	-	-	-	-	-	2/17	3/13	-	-	-	-
Schmolders J et al. [85]	2016	30	1	-	-	-	1/30	-	1/30	-	1/30	-	-	-
Schmolders J et al. [86]	2016	100	10	2/31	8/52	0/14	-	0/3	10/100	-	10/100	-	-	-
Donati F et al. [37]	2016	68	8	-	8/68	-	-	-	3/38	5/30	-	-	-	-
Pala E et al. [73]	2016	687	57	57/687	-	-	-	-	-	57/687	57/675	0/12 ***		
Calabró T et al. [28]	2016	109	6	-	6/109	-	-	-	-	6/109	6/109	-	-	-
Torner F et al. [102]	2016	7	1	1/6	0/1	-	-	-	-	1/7	1/7	-	-	-
Capanna R et al. [30]	2015	200	19	17/131	2/69	-	-	-	-	19/200	19/200	-	-	-
Pala E et al. [75]	2015	247	23	23/247	-	-	-	-	-	23/247	23/247	-	-	-
Ercolano LB et al. [39]	2015	282	31	15/15	11/11	3/3	-	2/2	-	31/282	10/10	16/16	-	5/5
Ueda T et al. [103]	2016	25	8	-	8/25	-	-	-	-	8/25	8/25	-	-	-
Mavrogenis AF et al. [64]	2013	225	27	27/225	-	-	-	-	-	27/225	27/225	-	-	-
Cho WH et al. [15]	2012	62	16	16/62	-	-	-	-	-	16/62	16/62	-	-	-
Ruggieri P et al. [82]	2012	669	60	60/669	-	-	-	-	-	60/669	60/669	-	-	-
Mavrogenis AF et al. [63]	2011	33	2	2/33	-	-	-	-	-	2/33	2/33	-	-	-
Bertani A et al. [25]	2009	23	3	-	3/23	-	-	-	-	3/23	2/15	-	-	1/8
Kostuj T et al. [58]	2013	68	15	6/32	2/18	7/17	-	0/1	-	15/68	2/22	13/46	-	-
Hardes J et al. [48]	2010	125	16	9/70	7/55	-	-	-	3/51	13/74	16/125	-	-	-
Mavrogenis AF et al. [65]	2015	1161	100	8/978	10/154	1/29	-	-	-	100/1161	100/1161	-	-	-
Toepfer A et al. [100]	2018	22	5	-	-	5/22	-	-	-	5/22	1/9	4/13 ***		
Puchner SE et al. [78]	2016	31	23	12/19	5/6	6/6	-	-	-	23/31	-	-	-	-
Ruggieri P et al. [81]	2010	16	5	5/16	-	-	-	-	-	5/16	5/16	-	-	-
Toepfer A et al. [99]	2016	18	8	-	-	8/18	-	-	-	8/18	-	3/11	-	5/7
Hussman B et al. [52]	2013	18	1	-	-	-	-	-	1/18	-	0/8	-	1/10	-
Höll S et al. [49]	2012	21	6	6/21	-	-	-	-	3/11	3/10	-	6/21 ***		
Cannon SR et al. [29]	2015	27	1	1/27	-	-	-	-	0/2	1/25	-	1/27	-	-
Lundh F et al. [61]	2014	17	3	2/10	1/5	0/2	-	-	-	3/17	-	3/17	-	-
Agarwal M et al. [19]	2010	26	2	2/26	-	-	-	-	-	2/26	-	2/26	-	-
Ribera J et al. [80]	2023	16	1	-	1/16	-	-	-	-	1/16	-	1/16 ***		
Fiore M et al. [40]	2023	45	8	8/45	-	-	-	-	8/45	-	-	-	8/45	-
Alvand A et al. [20]	2018	69	19	12/35	7/40	-	-	-	-	19/69	-	-	19/69	-
Marczak S et al. [62]	2017	9	1	1/9	-	-	-	-	-	1/9	-	0/7	1/2	-
De Gori M et al. [35]	2016	87	10	4/43	5/40	1/4	-	-	-	10/87	-	10/87 ***		
Glehr M et al. [41]	2013	31	4	-	-	-	-	-	4/31	-	-	-	-	-
Hu CC et al. [51]	2017	106	7	7/106	-	-	-	-	-	7/106	-	-	-	-
Scoccianti G et al. [87]	2016	33	3	0/19	2/13	1/1	-	-	3/33	-	-	-	-	-
Corona PS et al. [32]	2019	29	5	3/12	2/14	0/3	-	-	-	5/29	-	-	5/29	-
Sewell MD et al. [88]	2010	15	2	-	2/15	-	-	-	-	2/15	-	0/2	2/9	0/4
Artiaco S et al. [22]	2015	5	1	-	1/5	-	-	-	-	1/5	-	-	1/5	-
Crosby SN et al. [33]	2011	72	3	3/72	-	-	-	-	-	3/72	3/58	0/2	-	0/12
Tan PK et al. [97]	2009	19	6	-	-	-	-	-	-	6/19	6/19	-	-	-
Natarajan MV et al. [69]	2007	9	1	-	-	-	-	-	-	1/9	1/9	-	-	-
Bruns J et al. [27]	2007	25	1	-	-	-	-	-	-	1/25	1/25	-	-	-
Harde J et al. [48]	2010	20	1	0/3	1/9	-	0/5	0/3	1/20	-	1/20	-	-	-
Natarajan MV et al. [70]	2005	246	17	17/246	-	-	-	-	-	17/246	17/246	-	-	-
Müller P et al. [67]	2002	9	5	-	-	-	-	5/9	-	5/9	5/9	-	-	-
Ozaki T et al. [72]	2002	12	3	-	-	-	-	3/12	-	3/12	3/12	-	-	-
Ilyas I et al. [53]	2001	48	4	4/48	-	-	-	-	-	4/48	4/48	-	-	-
Parvizi J et al. [76]	2007	43	1	-	1/43	-	-	-	-	1/43	-	1/43 ***		
Springer BD et al. [94]	2004	26	5	5/26	-	-	-	-	-	5/26	-	5/26 ***		
Gosheger G et al. [42]	2001	69	6	3/15	3/33	0/5	0/16	-	-	6/69	6/69	-	-	-
Chandler H et al. [31]	1994	30	1	-	1/30	-	-	-	-	1/30	-	-	-	-
91		10,456	1277 (12%)	885/5780 (15%)	187/1402 (13%)	39/139 (28%)	13/142 (9%)	16/60 (27%)	141/1407 (10%)	1135/9049 (12.5%)	941/4283 (22%)	76/652 (12%)	84/318 (26%)	24/142 (17%)

* Infections/“lower limb” replacement; ** Infections/“upper limb” replacement; *** Infections/“non-oncological” (including trauma, PJI and mechanical failure).

## Data Availability

No new data were created in this study. Data sharing is not applicable to this article.

## Supplementary Materials

The following supporting information can be downloaded at: https://www.mdpi.com/article/10.3390/healthcare12131283/s1, The 27-item PRISMA checklist.
